# Electroacupuncture ameliorates pain in cervical spondylotic radiculopathy rat by inhibiting the CaMKII/CREB/BDNF signaling pathway and regulating spinal synaptic plasticity

**DOI:** 10.1002/brb3.3177

**Published:** 2023-08-07

**Authors:** Hong Su, Haiyan Chen, Xi Zhang, Shengyong Su, Jing Li, Yanjun Guo, Qiongxiao Wang, Caiyun Xie, Pu Yang

**Affiliations:** ^1^ The first school of clinical medicine, Guangxi of university of Chinese medicine Nanning China; ^2^ Department of nursing The First Affiliated Hospital of Guangxi University of Chinese Medicine Nanning China; ^3^ Department of Acupuncture and Moxibustion The First Affiliated Hospital of Guangxi University of Chinese Medicine Nanning China

**Keywords:** CaMKII/CREB/BDNF signaling pathway, central sensitization pain, cervical spondylotic radiculopathy, electroacupuncture, spinal cord synaptic plasticity

## Abstract

**Background:**

Central sensitization is one of the important mechanisms underlying neuropathic and radicular pain due to cervical spondylotic radiculopathy (CSR). Recent studies have shown that the calmodulin‐dependent protein kinase II (CaMKII)/cAMP‐response element binding protein (CREB)/brain‐derived neurotrophic factor (BDNF) signaling pathway mediates central sensitization through its involvement in spinal cord synaptic plasticity. Our group has previously found that electroacupuncture (EA) has a good analgesic effect on CSR. However, the central analgesic mechanism of EA for CSR is not yet clear.

**Methods:**

The rats were randomly divided into Blank group, Sham‐operated group, CSR group, and EA group. We prepared the CSR rat model using the fish wire extrusion method. The behavioral and mechanical pain thresholds of the rats in each group were measured 5 days after successful modeling and 7 days after the intervention. The first intervention was started 5 days after successful modeling, and the EA group was treated by acupuncture at the bilateral LI4 and LR3 points on the same side as one group, connected to a G6805‐I electroacupuncture apparatus with continuous waves at 1.5 Hz. The remaining groups were not subjected to EA intervention. The treatment was administered once a day for 7 consecutive days and then executed. We used WB, immunofluorescence, and qRT‐PCR to detect the expression of CaMKII/CREB/BDNF signaling pathway‐related factors in the synaptic of rat spinal cord in each group.

**Results:**

EA improved pain threshold and motor function in CSR rats, inhibited the expression of BDNF, P‐TrkB, CAMKII, and P‐CREB in spinal cord synapses, reduced the expression of pain factor c‐fos and postsynaptic membrane protein molecule neuroligin2, exerted a modulating effect on spinal cord synaptic plasticity in CSR rats, and suppressed the overactive synaptic efficacy.

**Conclusion:**

EA mediates central sensitization and exerts analgesic effects on CSR by modulating spinal synaptic plasticity, which may be related to the inhibition of CaMKII/CREB/BDNF signaling pathway.

## Graphical abstract

Electroacupuncture mediates central sensitization and exerts analgesic effects on CSR by modulating spinal synaptic plasticity, which may be related to the inhibition of CaMKII/CREB/BDNF signaling pathway.

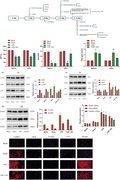

## 1 INTRODUCTION

Cervical spondylotic radiculopathy (CSR) refers to mechanical compression or inflammatory exudation, resulting in nerve root damage and clinical manifestations of neuropathic and radicular pain (Fujiwara et al., 2017; Study group on the standardization of diagnosis & treatment of neurogenic cervical spondylosis, Zhuang et al.,). Neuropathic and radicular pain due to CSR associated with peripheral and central sensitization. Local damage to the nerve root releases pro‐inflammatory cytokines, such as IL‐1β, IL‐6, and TNF‐α, causing inflammatory infiltration of the nerve root. This in turn increases the sensitivity of injurious receptor nerves to afferent signals and changes in ion channels, manifesting as peripheral sensitized pain (Von Hehn Christian et al., 2012). Persistent peripheral injurious stimuli can cause the release of excitatory neurotransmitters from the spinal cord. This causes hyperexcitation of spinal nerve roots and synaptic injurious sensory neurons, producing central sensitization (Zhu et al., 2019). Central sensitization is a complex pathological process involving spinal dorsal horn neurons, signaling pathways, synaptic plasticity, secondary to, and enhancing peripheral sensitization (Peirs et al., 2021). Spinal nerve roots and synaptic loops are key to integrating peripheral sensory information and inducing mechanical nociceptive responses. A growing body of evidence suggests that long‐term potentiation (LTP) of synaptic transmission plays an important role in the onset and maintenance of neuropathic or radicular pain (Lu et al., 2009). Increased postsynaptic Ca^2+^ inward flow in dendritic spines in the anterior cingulate cortex after nerve root injury induces LTP of synaptic transmission in the cingulate gyrus, resulting in abnormal pain in rats with nerve root injury (Bill & John, 2019). Central excitatory synaptic transmission is mainly mediated by glutamate (Glu), and *N*‐methyl‐d‐aspartic acid (NMDA) receptors are a Glu receptor subtype that enhances synaptic efficacy (Bliss et al., 2016). Damage to nerve roots activates NMDA receptors and alters presynaptic membrane potential. This in turn activates intracellular signaling pathways capable of initiating and maintaining central sensitization, producing pain sensitivity (D'Mello et al., 2011). Inhibition of this excitatory neurotransmitter expression reduces pain. Thus, modulation of spinal synaptic plasticity is an important therapeutic strategy for reducing neuropathic and radicular pain.

The calmodulin‐dependent protein kinase II (CaMKII)/cyclic adenosine monophosphate (cAMP)‐response element binding protein (CREB)/brain‐derived neurotrophic factor (BDNF) signaling pathway is involved in spinal synaptic plasticity. Ca^2+^/CaMKII is a regulator of central pain sensitization. It induces nociceptive sensitization by modulating synaptic transmission in neuronal excitatory and injurious sensory pathways (Fang et al., 2002). BDNF and its receptors are widely expressed in the nervous system, mainly in the central nervous system (Qian et al., 2023). BDNF binds to its specific receptor tyrosine kinase receptor B (TrkB) and activates the downstream signal phospholipase‐γ (PLC‐γ), which induces Ca^2+^/CaMKII binding and activates CaMKII. p‐CaMKII directly activates the synapse‐associated molecule CREB which CREB (Zhang et al., 2022). P‐CREB/CREB binding proteins (CBPs) bind to specific sequences of CRE on target genes and recruit RNA polymerase II to form transcriptional complexes, thereby regulating the transcription of target genes. The promoter regions that currently repress pain‐related genes are hyped in CRE sequences, including BDNF, c‐fos. P‐CREB regulates the transcription of BDNF and c‐fos and affects the synthesis of cytoskeleton‐associated and synaptic proteins, causing long‐lasting plasticity changes in spinal cord neurons and inducing pain maintenance and production (Wang et al., 2011). All of the above suggest the CaMKII/CREB/BDNF signaling pathway and central pain sensitivity are closely related.

Based on “regulation of peripheral sensitization,” our group has found EA can effectively relieve peripheral inflammatory pain in CSR rats (Chen et al., 2018a, 2018b; Huang, Su, Qin, et al., 2018; Huang, Su, Huang, et al., 2018; Zhao et al., 2018). The mechanism may be related to the inhibition of the expression of peripheral inflammatory factors IL‐1β, IL‐6, PGE2, and COX‐2 and the regulation of the JAK1/STAT3 signaling pathway. The peripheral analgesic mechanism of EA for CSR was initially demonstrated, but the mechanism of its modulation of the pain center is unclear. EA can inhibit central sensitization by suppressing excessive abnormal and adverse synaptic plasticity changes and improve the symptoms of abnormal nociceptive sensitization in rats with colitis and brachial plexus avulsion injury models (Pekny & Nilsson, 2005; Zhang et al., 2014). Based on the previous, this study further explores the central analgesic mechanism of EA for CSR from the perspective of modulating the primary center of pain and taking spinal synaptic plasticity as the entry point. The aim is to provide new ideas for the clinical treatment of CSR and contributes to the reduction of this recurrence rate and the improvement of patients’ quality of life.

## 2 MATERIALS AND METHODS

### 2.1 Laboratory animals and groups

**FIGURE 1:**
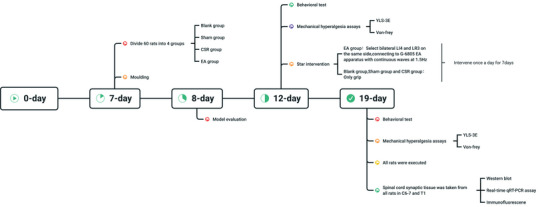
Time line of the experimental procedures.

A total of 60 adult male Sprague Dawley rats (SPF grade), weighing 250 ± 20 g, were provided by Changsha Tianqin Biotechnology Co., Ltd., Experimental animal Certificate No: SCXK (Xiang) 2019‐0014. Laboratory animals were kept at room temperature (20–25°C) with natural light illumination and diet ad libitum. According to the random numbers table, the experimental animals were randomly divided into four groups (remove the rats that failed to model): Blank group (*n* = 12), Sham group (*n* = 12), CSR model group (*n* = 12), and EA group (*n* = 12). All rats were used for behavioral and mechanical pain threshold testing 5 days post‐modeling and 7 days post‐intervention. All rats were executed 7 days post‐intervention and were used to detect the CaMKII/CREB/BDNF signaling pathway. The study protocol was conducted according to the Declaration of Helsinki protocol and the guidelines of the Laboratory Animal Care and Use Committee of the Laboratory Animal Center of Guangxi University of Chinese Medicine. This study was approved by the Animal Ethics and Welfare Committee of Guangxi University of Traditional Chinese Medicine (NO. DW20220430‐075). Figure time line of the experimental study is presented in Figure 1.

### 2.2 Model preparation

The CSR rat model was prepared using the cervical nerve root compression method using fish wire (Dou et al., 2006). All rats were fasted and dehydrated for 12 h prior to modeling. The rats were anesthetized with 2% sodium pentobarbital solution (0.2 mL/100 g) by intraperitoneal injection. They were fixed in prone position and the neck was prepared with a razor. After routine disinfection, the rat's highest cervical spine (T2) was first touched with the fingers. From there, an incision of approximately 3 cm in length was made upward in the midline of the neck. The subcutaneous tissues of the neck and the posterior cervical musculature are bluntly separated layer by layer to expose the left lateral arch of C6 to T2. The surface muscles and ligaments are then scraped away. The connective tissue and ligamentum flavum over the C6/C7 and C7/T1 intervertebral spaces are dissected with microforceps. The left vertebral arch of C7 is gently clamped open with a sharp‐billed mosquito forceps. A nylon fish wire (approximately 1.5 cm long and 0.5 mm in diameter) is placed under the nerve roots of C6 to T1 along the longitudinal axis of the spinal cord with microsurgical forceps and the nerve roots are compressed. The wound was closed layer by layer after placement. In the Sham‐operated group, no spinal canal insertion was performed and the rest of the procedure was performed as above.

Model evaluation: The rats were observed for autophagy, licking and biting, hissing, and other behaviors on the morning of the second day after modeling. Assessment of gait impairment due to painful contractures of their limbs in combination with the Kawakami method (Kawakami et al., 1994): 1 point if the rat had no deformity of the left forelimb and a normal gait; 2 points if the left forelimb has an inward foot curl deformity, slight weight holding or inability to hold weight, and walking limp; 3 points if the left forelimb had an inward foot curl deformity and walking without touching the table. A gait score of ≥2 was used as a criterion for successful modeling.

### 2.3 Electroacupuncture treatment

According to “Experimental Acupuncture Science” (Guo, 2016), the bilateral “He Gu” (LI4) and “Tai Chong” (LR3) are selected, with LI4 located between the first and second metacarpal bones of the forelimb, one point on each side. LR3 is located in the depression between the first and second metatarsal bones of the dorsal aspect of the hindlimb, one point on each side. Each point is needled for 20 min. During needle retention, the filiform needle handles at the ipsilateral LI4 and LR3 acupoints were connected to the G6805‐Ielectroacupuncture treatment device respectively and set to continuous wave. The frequency was 1.5 Hz (adjusted to the extent that the rat's muscles twitched lightly). The first EA treatment was performed 5 days after the animals were successfully molded, once a day for 7 days. Rats in the blank, Sham‐operated, and CSR model groups had the same grasping effect but were not given electroacupuncture treatment. Disposable sterile acupuncture needles (Hua Tuo, Suzhou Medical Products Co., Ltd.) were 13 mm in length and 0.25 mm in diameter. A G6805‐I electroacupuncture (EA) treatment device (Qingdao Xinsheng Industrial Co., Ltd.) was used. The product standard number was 20172270141.

### 2.4 Effect index and measurement method

#### 2.4.1 Behavioral test

Gait score: Before and after 7 days of intervention, the gait impairment caused by painful contracture of the rat limbs was assessed by referring to the Kawakami method (Kawakami et al., 1994), and the scoring criteria were the same as before.

#### 2.4.2 Mechanical hyperalgesia assays

The mechanical pain thresholds of the rats were measured before and after 7 days of the intervention using the **YLS‐3E** (Obata et al., 2004) electronic pressure pain meter. The pressure was gradually increased linearly by a blunt tip (Plexiglas cone) between the third and fourth metatarsal bones of the rat's left forefoot until the rat screamed and struggled to pull back the foot. The pressure value (g) recorded automatically by the apparatus at this point was the mechanical pain threshold of the rat and was averaged over three consecutive measurements.

The assessment was carried out using the Von Frey method, in which (0.4, 0.6, 1.0, 1.4, 2.0, 4.0, 6.0, 8.0, and 15.0 g) Von Frey filaments were used to vertically stimulate the skin of the rat's left upper limb at mid‐palm starting at 2.0 g strength. The stimulation time was 6–8 s each time and the rats’ responses were observed sequentially. If the rat shows a foot retraction or licking response after stimulation, the rat is considered positive and marked with (×). If there is no response, the rat is considered negative and is marked with (○). The animals should be stimulated at least 10 min apart from each other. The second experiment is performed more than 4 days apart. The presence of NP was considered when the Von Frey monofilament stimulation intensity was less than 4 g.

#### 2.4.3 Western blot

Weigh 50 mg of rat spinal cord tissue, cut and ground in liquid nitrogen to a fine powder, divided into centrifuge tubes, added 500 μL of RIPA lysis solution, mixed thoroughly, extracted protein, and determined the protein concentration. The gels were denatured at 95°C for 10 min; SDS–PAGE gels were prepared, sampled, electrophoresed, transferred, and closed with 5% skimmed milk powder for 2 h–4°C reaction overnight incubation of primary antibody CAMKII (1:1000, Abcam), TRKB (1:1000, Affinity), P‐TRKB (1:1000, Affinity), CREB (1:1000, Affinity), P‐CREB (1:1000, Affinity), C‐FOS (1:800, Wuhan Sanying), BDNF (1:1200, Abcam), and neuroligin2 (NLGN2) (1:1200, Affinity). The membrane was then washed, followed by incubation of the secondary antibody (1:3000), and the membrane was washed again. The ECL reagent was mixed with liquid A and liquid B in the ratio of 1:1, and the mixture was evenly dropped onto the membrane, spread with cling film and wrapped with PVDF film, and put into the dark box, respectively, and exposed for development. Finally, the gray scale values of the strips were measured by ImageJ software, and the ratio of the obtained gray scale values to the gray scale values of the internal reference was used as the relative expression of the measured proteins.

#### 2.4.4 Real‐time qRT‐PCR assay

Rat spinal cord tissue of 100 mg was weighed, total RNA was extracted by Trizol method, and the concentration and quality of RNA were measured by a DeNovix ultramicro UV–visible spectrophotometer. A amount of 1‐μg RNA was reverse transcribed into cDNA, which was used as a template for real‐time PCR amplification. Reaction procedure: pre‐denaturation, 95°C, 15 min, 1 cycle; denaturation, 95°C, 10 s; annealing, 58°C, 30 s; extension, 72°C, 30 s; 40 cycles in total. The primer sequences are shown in Table 1. Fluorescence quantitative PCR instrument was used for the detection of gene expression, and melting curve analysis was performed at 60–95°C. The relative expression of target mRNAs was calculated by the 2^−△△^ *^Ct^* method using β‐actin as an internal reference.

**TABLE 1:** Oligonucleotide sequences of primers.

Gene	Upstream	Downstream
BDNF	GCCTCCTCTGCTCTTTCTGC	TTTATCTGCCGCTGTGACCC
CAMKII	CCAATATCGTCCGACTCCA	GGCATCAGCCTCACTGTAAT
TRKB	GGGCTTATGCTTGCTGGTCT	TCTGGGTCAATGCTGTTAGGT
β‐Actin	AGATTACTGCCCTGGCTCCTAG	CATCGTACTCCTGCTTGCTGAT

#### 2.4.5 Immunofluorescence

The tissue sections were dewaxed to water and repaired under high pressure with 3% citric acid. Wash with distilled water for 5 min, add 3% hydrogen peroxide for 10 min, then wash with distilled water for 5 min and soak in PBS for 1 min. After incubation with 5% BSA for 30 min at room temperature, BSA was shaken off (do not wash), and the primary antibody working solution was added dropwise overnight at 4°C. After returning to room temperature, the sections were removed the next day and washed three times for 5 min each with PBS buffer. Fluorescent secondary antibody was added dropwise and incubated for 30 min at 37°C; PBS buffer was washed three times for 5 min each time; DAPI was added dropwise and incubated for 10 min at room temperature, shabu–shabu washed with tap water, and then the tablets were sealed with water‐soluble sealer. The distribution and content of CaMKII, CREB, P‐CREB, NLGN2, and c‐fos in the cells were observed by fluorescence microscopy. Finally, the average absorbance values of CaMKII, CREB, P‐CREB, NLGN2, and c‐fos protein positive expression in all samples were measured by ImageJ.

### 2.5 Statistical analysis

SPSS 20.0 software was used for statistical analysis. The measurement data were expressed as mean ± standard deviation, in accordance with chi‐square, and one‐way ANOVA was used for comparison between multiple groups, and LSD test was used for two‐way comparison between groups; Kruskal–Wallis rank sum test was used for comparison between multiple groups for rank data. Differences were considered statistically significant at *p* < .05.

## 3 RESULTS

### 3.1 Electroacupuncture increased the mechanical pain threshold in CSR rats

**FIGURE 2:**
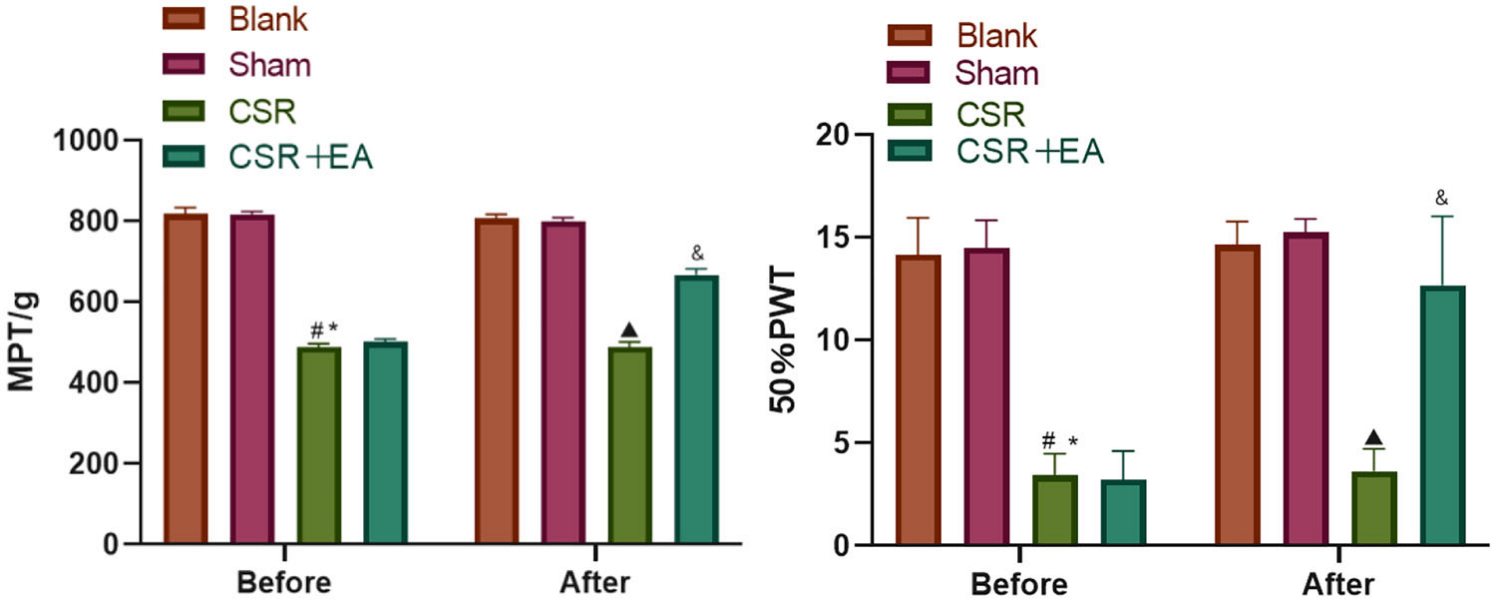
Pain threshold rats before and after intervention. Before intervention ^#^ *p* < .05, versus Sham group; ^*^ *p* > .05, versus CRS + EA group. After intervention ^▲^ *p* < .05, versus Sham group; ^&^ *p* < .05, versus CRS group. CSR, cervical spondylotic radiculopathy; CSR + EA, cervical spondylotic radiculopathy + electroacupuncture.

The results are presented in Figure 2. Relative to the Sham group, the pain thresholds of the CSR and EA groups were significantly reduced before the intervention (*p* < .05). After the intervention, the pain threshold in the EA group relative to that of the CSR group increased distinctly (*p* < .05).

### 3.2 Electroacupuncture improved the behavioral scores in CSR rats

**FIGURE 3:**
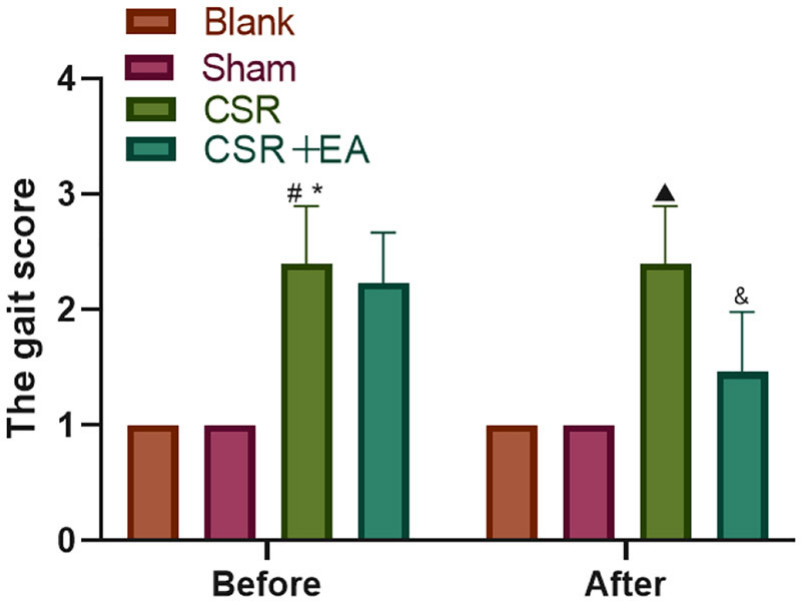
Motor function of rats before and after intervention. Before intervention ^#^ *p* < .05, versus Sham group; ^*^ *p* > .05, versus CRS + EA group. After intervention ^▲^ *p* < .05, versus Sham group; ^&^ *p* < .05, versus CRS group. CSR, cervical spondylotic radiculopathy; CSR + EA, cervical spondylotic radiculopathy + electroacupuncture.

The results are presented in Figure 3. The gait scores for the CSR and EA groups were markedly higher prior to intervention (*p* < .05) than those of the Sham group. Relative to the CSR group, the EA group showed a significant decrease in gait scores after intervention (*p* < .05).

### 3.3 Electroacupuncture inhibited the central sensitization by mediating the BDNF/TrkB signaling pathway in the spinal cord of CSR rats

**FIGURE 4:**
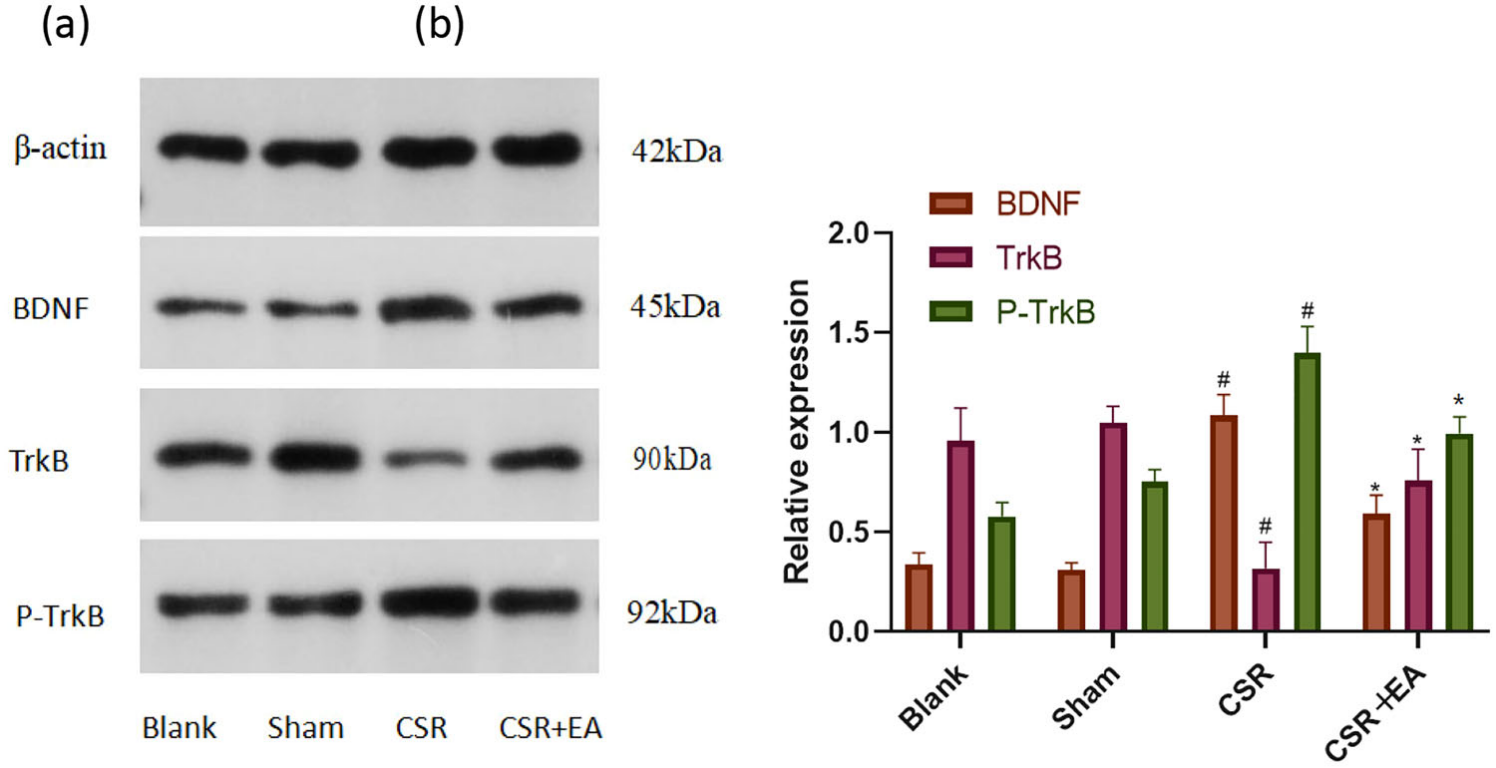
Brain‐derived neurotrophic factor (BDNF), tyrosine kinase receptor B (TrkB), and P‐TrkB protein expression in the spinal dorsal horn at 7 days after intervention. (a) Western blot assay of BDNF, TrkB, and P‐TrkB protein. (b) Relative protein band densities of BDNF, TrkB, and P‐TrkB. The densities of the protein bands were analyzed and normalized to β‐actin. ^#^ *p* < .05, versus Sham group; ^*^ *p* < .05, versus CRS group. CSR, cervical spondylotic radiculopathy; CSR + EA, cervical spondylotic radiculopathy + electroacupuncture.

After the completion of behavioral tests, the expression of BDNF, TrkB, and P‐TrkB in the spinal cord synaptic tissue of rats in each group was detected by WB and PCR. As shown in Figures 4 and 8, BDNF, P‐TrkB protein and BDNF mRNA, and TrkB mRNA expression were increased (*p* < .01), and TrkB expression was decreased (*p* < .01) in the spinal cord tissue of rats in the CSR group compared with the Sham group. Compared with the CSR group, the expressions of BDNF, P‐TrkB protein and BDNF mRNA, and TrkB mRNA were decreased (*p* < .01), and TrkB expression was increased (*p* < .01) in the spinal cord tissue of the EA group rats. These results suggest that EA can reduce the autophosphorylation of TrkB protein and inhibit the transduction of BDNF/TrkB pathway in the spinal cord tissue of CSR rats.

### 3.4 Electroacupuncture regulated synaptic plasticity mediated by inhibiting CAMKII/CREB signaling pathway in the spinal cord of CSR rats

**FIGURE 5:**
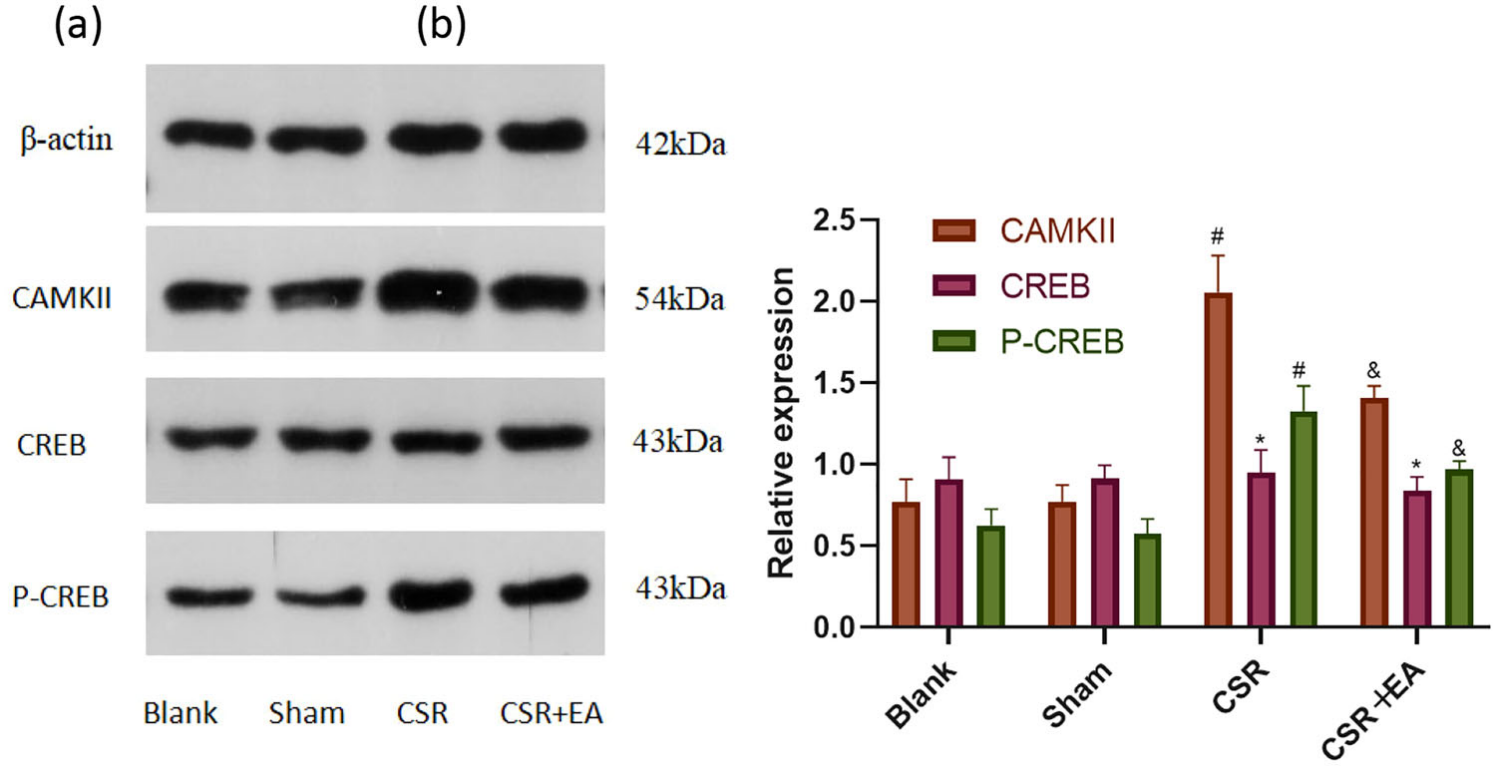
Calmodulin‐dependent protein kinase II (CAMKII), cAMP‐response element binding protein (CREB), and P‐CREB protein expression in the spinal dorsal horn at 7 days after intervention. (a) Western blot assay of CAMKII, CREB, and P‐CREB protein. (b) Relative protein band densities of CAMKII, CREB, P‐CREB. The densities of the protein bands were analyzed and normalized to β‐actin. ^*^ *p* > .05, versus Sham group; ^*^ *p* > .05, versus CRS group; ^#^ *p* < .05, versus Sham group; ^&^ *p* < .05, versus CRS group. cAMP, cyclic adenosine monophosphate; CSR, cervical spondylotic radiculopathy; CSR + EA, cervical spondylotic radiculopathy + electroacupuncture.

The expressions of CAMKII, CREB, and P‐CREB in the spinal cord tissues were detected by WB, PCR, and immunohistochemistry. The results are presented in Figures 5, 7, and 8, there was no significant difference in the expression of CREB in the spinal cord tissues of the spinal cord among the four groups(*p* > .05). The expression of CAMKII, CAMKII mRNA, and P‐CREB was increased in the spinal cord synaptic tissue of rats in the CSR group compared with the Sham group (*p* < .01). The expression of CAMKII, CAMKII mRNA, and P‐CREB was reduced in the spinal cord tissue of rats in the EA group compared with the CSR group (*p* < .01). Immunofluorescence analysis showed that the staining density of CAMKII was weaker in the EA group compared with the CSR group, but no significant difference was seen in the staining density of CREB and P‐CREB in each group. The above suggests that EA inhibits CAMKII/CREB pathway transduction in the spinal cord tissue of CSR rats.

### 3.5 Electroacupuncture reduced the release of pain factors in the spinal cord synaptic tissue of CSR rats

**FIGURE 6:**
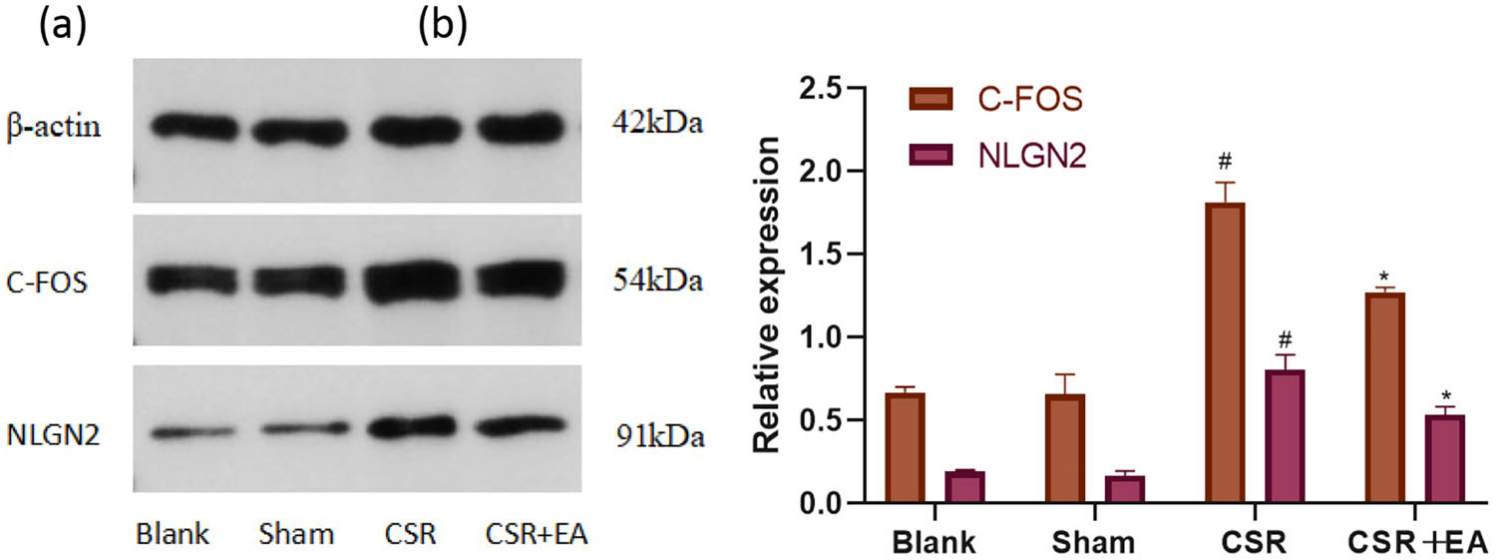
c‐fos, neuroligin2 (NLGN2) protein expression in the spinal dorsal horn at 7 days after intervention. (a) Western blot assay of c‐fos, NLGN2 protein. (b) Relative protein band densities of c‐fos, NLGN2. The densities of the protein bands were analyzed and normalized to β‐actin. ^#^ *p* < .05, versus Sham group; ^*^ *p* < .05, versus CRS group. CSR, cervical spondylotic radiculopathy; CSR + EA, cervical spondylotic radiculopathy + electroacupuncture.

**FIGURE 7:**
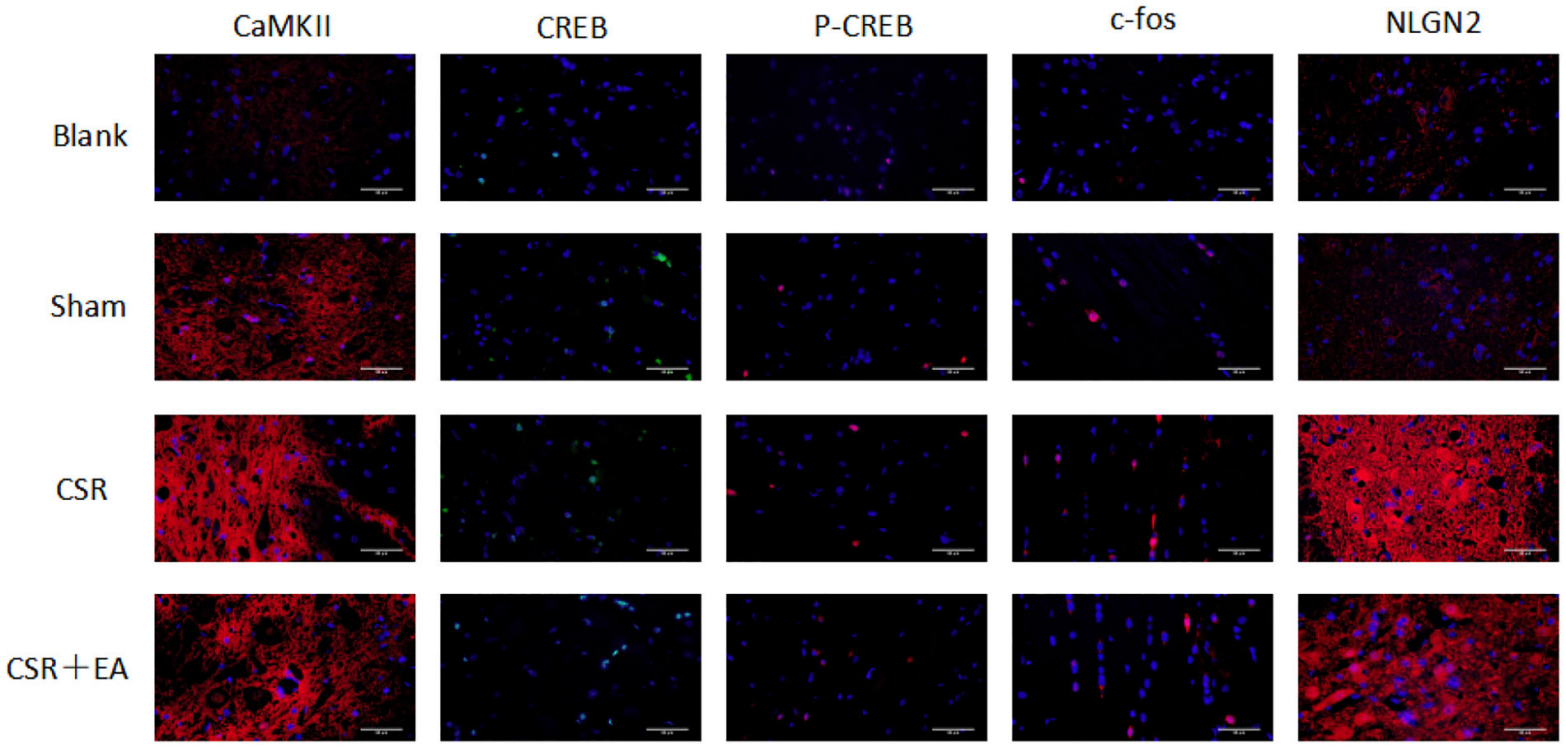
Immunofluorescence staining of calmodulin‐dependent protein kinase II (CAMKII), cAMP‐response element binding protein (CREB), P‐CREB and c‐fos, neuroligin2 (NLGN2) expression in the spinal dorsal horn at 7 days after intervention. Representative CAMKII, CREB, P‐CREB and c‐fos, NLGN2 photomicrographs from the spinal dorsal horn at 7 days after intervention. DAPI‐stained nuclei are blue under UV excitation, with positive expression as red or green light labeled with the corresponding fluorescein. cAMP, cyclic adenosine monophosphate; CSR, cervical spondylotic radiculopathy; CSR + EA, cervical spondylotic radiculopathy + electroacupuncture.

c‐fos can be used as a marker of neuronal excitation. We used WB and immunofluorescence method to detect c‐fos expression in the spinal cord tissues of rat spinal cord in each group. The results are presented in Figures 6 and 7, the expression of c‐fos expression was elevated in spinal cord synaptic tissue of rats in the CSR group compared with the Sham group (*p* < .05), the expression of c‐fos was reduced in the spinal cord tissues of rat spinal cord in the EA group compared with the CSR group (*p* < .05). Immunofluorescence analysis showed that the staining density of c‐fos was weaker in the EA group compared to the CSR group. The above suggests that EA can inhibit the expression of c‐fos, the pain target gene of CRE sequence promoter, and inhibit the excitation of spinal cord synaptic neurons, which in turn has an analgesic effect.

**FIGURE 8:**
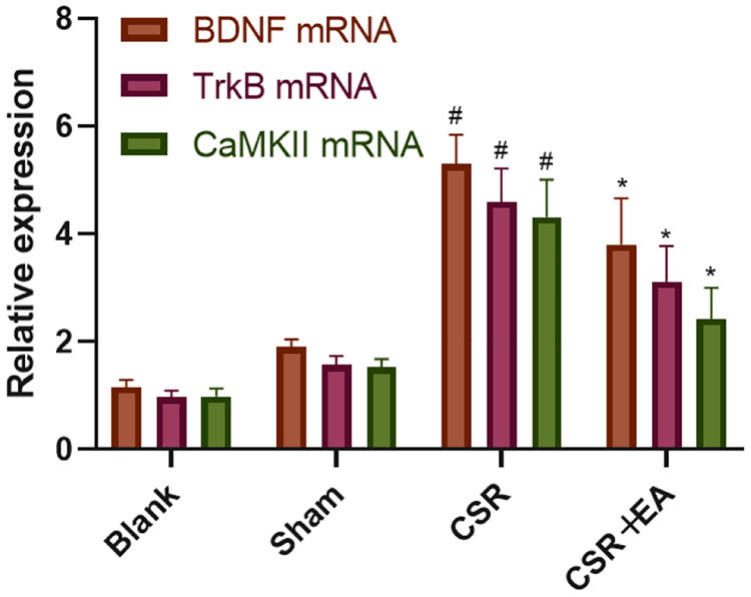
Brain‐derived neurotrophic factor (BDNF) mRNA, tyrosine kinase receptor B (TrkB) mRNA, and calmodulin‐dependent protein kinase II (CAMKII) mRNA expression in the spinal dorsal horn at 7 days after intervention. ^#^ *p* < .05, versus Sham group; ^*^ *p* < .05, versus CRS group. CSR, cervical spondylotic radiculopathy; CSR + EA, cervical spondylotic radiculopathy + electroacupuncture.

### 3.6 Electroacupuncture decreased the expression of postsynaptic membrane factors in the spinal cord synaptic tissue of CSR rats

NLGN2 has a role in regulating neuronal cell shape and synaptic plasticity and can be used to respond to synaptic structural or functional activity. We used WB and immunofluorescence method to detect NLGN2 expression in the synaptic tissue of rat spinal cord in each group. The results showed in Figures 6 and 7, the expression of NLGN2 was increased in the spinal cord synaptic tissue of rats in the CSR group compared with the Sham group (*p* < .05), NLGN2 expression was reduced in the spinal cord synaptic tissue of rats in the EA group compared with the CSR group (*p* < .05). Immunofluorescence analysis showed that the staining density of NLGN2 was weaker in the EA group compared to the CSR group. Therefore, we suggest that EA ameliorates poor or overactive synaptic activity by inhibiting NLGN2 expression in spinal cord, which in turn inhibits nociceptive sensitization in CSR rats.

## 4 DISCUSSION

Central sensitization is one of the key mechanisms mediating pain symptoms in CSR. Changes in spinal synaptic plasticity mediate the CaMKII/CREB/BDNF signaling pathway, causing central sensitization (Zhou et al., 2022). CaMKII is a regulator of peripheral sensitization and central sensitization. It is distributed in the pain information processing regions of the superficial dorsal horn and dorsal root ganglia of the spinal cord. CREB is an important downstream signaling molecule of CaMKII. It can regulate nociceptive genes by regulating the morphological plasticity of dendritic spines. Activation of Ca^2+^/CaMKII in the spinal cord induces CREB phosphorylation and P‐CREB binds to CBP. It binds to a specific sequence of CRE on the target gene and recruits RNA polymerase II to form a transcriptional complex, which in turn regulates the transcription of the target gene. CRE sequences are known to be present in the promoter regions of many pain‐related genes, including c‐fos and BDNF. c‐fos and BDNF expressions are mediated by CREB in response to injurious stimuli, which in turn causes long‐lasting plasticity changes in spinal cord neurons and is involved in pain chronicity (Yan et al., 2016). BDNF slows down the firing rate of inhibitory neuronal action potentials and reduces the frequency of spontaneous inhibitory postsynaptic currents in the dorsal horn of the spinal cord, thereby inhibiting downstream inhibition (Ding et al., 2015). Previous studies have found that reducing or inhibiting BDNF expression reduces neuropathic pain (Khan et al., 2015). BDNF binds to TrkB receptors, inducing receptor TrkB coupling and autophosphorylation. TrkB phosphorylation activates PLC‐γ, which induces Ca^2+^/CaMKII binding and thus activates the CaMKII/CREB/BDNF pathway. Increased BDNF expression leads to massive Glu release, which activates postsynaptic ionotropic NMDA. Peripherally stimulated sustainable activation of NMDA receptors and increased Ca^2+^ influx. Then it excites neurons and enhances the excitatory effect of pain transduction (Huang et al., 2022). NLGN2 is a postsynaptic membrane molecule that is closely related to synaptic transmission. Nociceptive stimulation enhanced NLGN2 binding to postsynaptic density‐95 in the spinal cord of mice. Furthermore, it regulates synaptic targeting of the α‐amino‐3‐hydroxy‐5‐methyl‐4‐isoazolepropionic acid (AMPA) receptor subtype GluR1 and involves in the formation of pain (Guo et al., 2018; Kim Ji‐Young et al., 2016). These results suggest that the CaMKII/CREB/BDNF signaling pathway is closely linked to central sensitization.

EA can inhibit or reduce central sensitization and relieve pain by modulating spinal synaptic plasticity. LTP is an important manifestation of synaptic plasticity and is closely related to modulation of pain transmission (Ji et al., 2018). After L5‐6 spinal nerve ligation and local nerve root entrapment, rats exhibit mechanical nociceptive sensitization and induce LTP effects in class C nociceptive conduction fibers at the dorsal horn of the spinal cord. EA ameliorated adverse synaptic plasticity changes and induced long‐term depression (LTD) effects in the dorsal horn of the spinal cord in rats. A reduction in the expression of inhibitory neurotransmitters γ‐aminobutyrate and BDNF in the dorsal horn of the spinal cord was also observed (Zheng et al., 2020). In addition, EA can reduce central sensitization and improve symptoms of abnormal nociceptive sensitization in rats with colitis and brachial plexus avulsion injury models by inhibiting excessive abnormal, adverse synaptic plasticity changes (Pekny & Nilsson,). It was found that EA inhibited BDNF and TrkB expression in the dorsal horn of the spinal cord of rats and complete Freund's adjuvant rats after chronic compressive injury to the sciatic nerve and increased pain thresholds (Yuan, 2021; Zhang et al., 2018). Ma's study found that EA inhibited P‐CREB and P‐ERK expression in the dorsal horn of the spinal cord of rats with diabetic neuralgia (Ma et al., 2022). It has been demonstrated that EA inhibits the expression of CaMKII, BDNF mRNA, TrkB mRNA, cyclic adenosine monophosphate mRNA and CREB mRNA in the dorsal horn of the spinal cord of rats with painful cervical incisions (Lin et al., 2012; Qiao et al., 2017; Wang et al., 2012). Our group's previous study also confirmed that EA can reduce CSR‐induced pain by inhibiting peripheral sensitization. This study will further extend the previous study by moving from the peripheral analgesic mechanism of EA to the central analgesic mechanism. The possible central analgesic mechanisms of EA for pain relief in CSR will be systematically explored.

Nociceptive sensitization can result from both the incision of the CSR mold and the nerve roots trapped by the fish wire. The pain in the surgical opening is acute and can develop into neuropathic pain. Therefore, we set up a Sham‐operated group and a Blank group in order to exclude the interference caused by the pain of the operative opening. In our study, we found that the mechanical pain threshold of the Sham‐operated group was lower than that of the Blank group, but there was no statistical difference (*p* > .05). The above suggests that the nociceptive sensitivity of the CSR and EA groups was mainly caused by the nerve root entrapment by the fish wire. The fish wire entrapment on cervical nerve roots was persistent, and CaMKII expression in the spinal cord tissue of rats in the model group was elevated and mechanical pain thresholds were lowered, suggesting that Ca^2+^/CaMKII was activated in the spinal cord. Therefore, we examined the synaptic‐related molecule CREB downstream of CaMKII and found no significant difference in CREB protein expression in the spinal cord tissues of the rats in each group. However, the expression of P‐CREB in the spinal cord tissues of the model group was higher than that of the Sham‐operated and EA groups, suggesting that CREB phosphorylation was activated after CSR. On the one hand, we detected elevated BDNF and P‐TrkB/TrkB in the spinal cord tissue of the CSR group. On the other hand, we found elevated expression of the pain gene promoter c‐fos in spinal cord tissues of the CSR group. The above suggests that CREB phosphorylation is involved in and maintains pain by mediating two pathways, BDNF and c‐fos. There is a bidirectional regulatory relationship between CREB and BDNF. Binding of BDNF to TrkB activates the PLC‐γ pathway, which can further induce Ca^2+^/CaMKII binding and activate CaMKII/CREB. Furthermore, the downstream pain target gene c‐fos transcription is accelerated, which can affect the synthesis of cytoskeleton‐associated and synaptic proteins, causing long‐time course plasticity changes in spinal cord neurons (Stanislava et al., 2008). After EA intervention, the expression of CAMKII, P‐CREB, c‐fos, BDNF, and P‐TrkB/TrkB in the spinal cord decreased, and pain and gait scores improved compared to before. To further validate the modulation of synaptic plasticity in the spinal cord of rats with CSR, we examined NLGN2 in the spinal cord of rats, whose connection to PSD95 modulated the synaptic targeting of the AMPA receptor subtype GluR1. NLGN2 expression was increased in the spinal cord of rats after modeling, suggesting abnormal synaptic activity. After EA intervention, NLGN2 expression in the spinal cord was reduced. Our study indirectly verified the regulation of synaptic plasticity in the spinal cord of CSR rats by observing the inhibition of the CaMKII/CREB/BDNF signaling pathway by EA.

We believe that persistent pain can cause structural changes in the brain, which has inspired our clinical research. The real‐time integration of neuronal activity in the brain, influenced by interactions between different brain regions or nuclei, is involved in maintaining pain. On the one hand, brain structures, such as the insula, cingulate gyrus, prefrontal cortex, thalamus, and spinal cord, are all involved in nociceptive information generation (Li et al., 2022). We can observe the modulation of the neural response pattern of the brain network in CSR patients by EA treatment through multimodal nuclear magnetic resonance and electroencephalography information data acquisition. Further, alterations in brain structure can bring about mania, mental depression, sleep disorders, learning and memory impairment, and personality deficits. Knockdown of the Ca^2+^ binding protein Kv channel interacting protein 3 gene in rats has been shown to induce pain‐related intense aversion, anxiety, and depression (Tian et al., 2018). A growing body of clinical and basic research suggests that pain is closely associated with abnormalities in the function of neural structures involved in emotion and motivation (Liu, 2022). Acupuncture can both reduce pain and improve the negative emotions associated with pain by modulating the brain's reward circuit (Zhang et al., 2012). The brain mechanisms of non‐opioid analgesia and related drug development are hot and difficult issues in the field of pain. It has been shown that altered neuronal activity in the reward circuit of the brain can affect the synthesis and release of neuropeptides such as BDNF and Glu to downstream brain regions, thereby mediating pain sensitivity and affective disorders (Zhang et al., 2015). It is suggested that acupuncture has the potential to be involved in non‐opioid analgesia. Therefore, exploring the brain reward loop and related molecular mechanisms of acupuncture analgesia has the potential to alleviate the current growing global opioid crisis.

However, our study still has some limitations. First, we confirmed that EA inhibits central sensitization in relation to the CaMKII/CREB/BDNF signaling pathway, lacking the detection of LTP and LTD signaling in class C nociceptive fibers at the dorsal horn of the spinal cord. Second, we confirmed that EA modulates synaptic functional plasticity and lacked observations of altered synaptic structure. Despite these limitations, the present study provides a more solid theoretical basis for the treatment of pain with EA than previous studies by elucidating the mechanism of action of EA in the treatment of CSR from the perspective of “mediating central sensitization.”

### PEER REVIEW

The peer review history for this article is available at https://publons.com/publon/10.1002/brb3.3177.

## Data Availability

The data that support the findings of this study are available from the corresponding author upon reasonable request.
